# Prevalence and Management of Alkyl-Methoxypyrazines in a Changing Climate: Viticultural and Oenological Considerations

**DOI:** 10.3390/biom11101521

**Published:** 2021-10-15

**Authors:** Gary J. Pickering, Jim Willwerth, Andreea Botezatu, Margaret Thibodeau

**Affiliations:** 1 Department of Biological Sciences, Brock University, St. Catharines, ON L2S 3A1, Canada; jwillwerth@brocku.ca (J.W.); mthibodeau@brocku.ca (M.T.); 2 Department of Psychology, Brock University, St. Catharines, ON L2S 3A1, Canada; 3 Cool Climate Oenology and Viticulture Institute, Brock University, St. Catharines, ON L2S 3A1, Canada; 4 National Wine and Grape Industry Centre, Charles Sturt University, Wagga Wagga, NSW 2678, Australia; 5 Sustainability Research Centre, University of the Sunshine Coast, Sippy Downs, QLD 4556, Australia; 6 Department of Horticultural Sciences, Texas A&M University, College Station, TX 77843-2133, USA; abotezatu@tamu.edu

**Keywords:** grape secondary metabolites, wine, methoxypyrazines, climate change, ladybug taint, climate adaptation

## Abstract

Alkyl-methoxypyrazines are an important class of odor-active molecules that contribute green, ‘unripe’ characters to wine and are considered undesirable in most wine styles. They are naturally occurring grape metabolites in many cultivars, but can also be derived from some Coccinellidae species when these ‘ladybugs’ are inadvertently introduced into the must during harvesting operations. The projected impacts of climate change are discussed, and we conclude that these include an altered alkyl-methoxypyrazine composition in grapes and wines in many wine regions. Thus, a careful consideration of how to manage them in both the vineyard and winery is important and timely. This review brings together the relevant literatures on viticultural and oenological interventions aimed at mitigating alkyl-methoxypyrazine loads, and makes recommendations on their management with an aim to maintaining wine quality under a changing and challenging climate.

## 1. Introduction

3-Alkyl-2-Methoxypyrazines (MPs) are a group of volatile, nitrogen-containing heterocyclic compounds found throughout the natural world, including plants and insects [1,2]. MPs are significant aroma compounds in a wide range of important grapevine cultivars in the global grape and wine industry. MPs are generally associated with vegetative tissues and immature fruit [1]. When present, they contribute to the vegetal (e.g., bell pepper, peas, leafy), earthy, nutty, and/or moldy aroma of a wine [3]. MPs are key aromatic compounds in some grape cultivars, including Sauvignon blanc [4,5], Cabernet Sauvignon [6], Cabernet franc [7], Merlot [8], and Carménère [9], where low levels contribute to the varietal character of these wines. However, MPs are generally undesirable and are considered fault compounds when they are present at high levels in wines [10].

**Figure 1:**
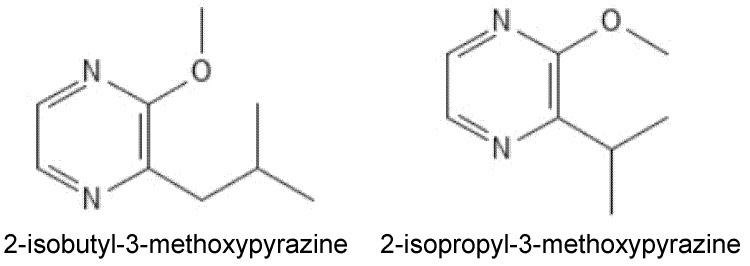
The 2-isobutyl-3-(IBMP) and 2-isopropyl-3-(IPMP) methoxypyrazine are the most prevalent methoxypyrazines found in grapes and wine affected by ladybug taint, respectively.

The main endogenous MP found in grapes is 3-isobutyl-2-methoxypyrazine (IBMP). In contrast, sec-butyl-2-methoxypyrazine (SBMP), 3-isopropyl-2-methoxypyrazine (IPMP), and 2,5-dimethyl-3-methoxypyrazine (DMMP) can be present endogenously at lower levels in grapes or introduced exogenously into wines through fruit contaminated with beetles from the Coccinellidae family [11] (Figure 1). MPs are extremely potent odorants, typically detected in grapes and wines at parts per trillion (ng/L; [12]). Specific human sensory thresholds in wine have been reported as IBMP, 5–16 ng/L [13,14]; SBMP, 1–2 ng/L (in water, [13]); IPMP, 0.3–2 ng/L [15,16]; DMMP, 31 ng/L [17]. Common with many odor molecules, individuals show a significant variation in their sensitivity to MPs, and detection thresholds vary with wine style [16]. Consumer rejection thresholds for IBMP have been reported for Sauvignon (50 ng/L) and Fer (30 ng/L) [14], suggesting that IBMP may be acceptable in some wine styles. Consumer rejection thresholds have not been reported for wine styles where MPs do not contribute to varietal typicity, nor for IPMP, SBMP, or DMMP in any wine style. Given their potency and impact on wine quality, the MP content of wine needs to be controlled through appropriate decisions both in the vineyard and the winery.

The synthesis, degradation, and final concentration of endogenous MPs in grape berries are highly impacted by the grapevine cultivar and climate [18,19,20]. Furthermore, a changing climate is also implicated in the spread and survival of Coccinellidae, an exogenous source of MPs, in winemaking regions across the globe [21]. Therefore, it is both important and timely to consider the impact of climate change on the MP composition of grapes and wine, and how MPs can best be managed to optimise wine quality.

## 2. Endogenous Methoxypyrazines

### 2.1. Distribution of MPs

The location of MPs in grapevine tissues is an important consideration when understanding how to manage them both in the vineyard and the winery. MPs are found throughout the entire grapevine, including the grape berries, cluster rachis, leaves, shoots, and roots [22,23]. The form and concentration of MPs may vary depending on the grapevine part. For example, very high concentrations of MPs are found in grape roots (8000 ng/L), and these are primarily IPMP, whereas IBMP dominates in the berries, leaves, and shoots [24,25]. Older leaves have the highest IBMP concentrations, and they increase during grapevine growth as the season progresses [25]. In comparison, clusters, young leaves, and lateral shoots contain lower amounts of IBMP [25]. An analysis of the distribution of MPs in grape berries indicates that 72% of IBMP is found in the skin, 23.8% in the seeds and only 4.2% of IBMP in the pulp [25]. Most (79.2%) of the MPs in grape clusters are located in the stems [25]. It is, therefore, necessary to reduce material, other than grapes, (MOG) such as rachises, petioles, and leaves, for a high-quality wine production [26]. In the vineyard, modern harvesting equipment, including optical sorting technology, can help reduce MOG with a high MP concentration from entering harvest bins and/or fermentation tanks, thereby reducing the overall level of MPs and other undesirable compounds in the finished wines [27,28].

### 2.2. Accumulation and Degradation of MPs 

In grape berries, IBMP accumulates in a double sigmoidal pattern prior to veraison (i.e., the onset of berry ripening). IBMP concentration increases in developing berries between fruit set and then again 2–3 weeks before the onset of veraison [19]. This is followed by a rapid decrease in concentration post veraison [19]. An important factor in MP accumulation in grape berries is the enzymatic methylation of hydroxypyrazine to MPs by *O*-methyltransferases [22,29]. Dunlevy et al. [29] found an increase in expression of genes that encode some *O*-methyltransferase enzymes between 4- and 8-weeks post-flowering, which coincided with the accumulation of MPs in these berries, with expression levels then declining post-veraison [22,29]. The biosynthesis of MPs is influenced by sunlight [22,30,31] and, in several studies, exposing berries to light increased the concentration of MPs in immature berries [31,32]. Some research supports the theory that IBMP can be transported from the leaves to the berries [25]. However, the biosynthetic pathways of MP synthesis are largely unknown, and the metabolism of MPs may differ between grape berries and other grapevine tissues [33]. 

MPs decrease rapidly post-veraison through to harvest [19]. Photodegradation via sunlight was once thought to be the main mechanism for the decrease in MPs in grapes [34,35]. However, more recent studies suggest that this is not the case [13,32] and that the decrease in IBMP is temperature-dependent. Warmer temperatures, particularly at night, can decrease IBMP more rapidly along with malic acid [9]. Degradation is likely due, at least in part, to the *O*-demethylation enzymatic pathway(s) [3]; however, MP degradation in general is not well understood [23]. The final concentration of MPs in grapes is the result of a balance between the biological formation and degradation of MPs throughout the maturation process in berries.

### 2.3. Impact of Climate Change

MP content in wines depends largely on their concentration in the grapes at harvest. As grapevine maturation is largely governed by environmental factors [36], climate can have a profound impact on the balance between MP synthesis and degradation [37,38].

MPs form largely in the earlier stages of grape development and may be higher in the ripe fruit when temperatures are lower [18,38]. Thus, cool ripening conditions may lead to higher MP levels [32,34,39]. In contrast, multiple studies have demonstrated that wines from warmer regions have lower IBMP levels and less characteristics associated with MPs, such as vegetative and herbaceous flavours [12,18,40]. In addition, Belancic and Agosin [9] observed that a higher proportion of days over 30 °C resulted in vintages with the lowest MP concentrations in Carménère grown in Spain. Thus, temperature plays an important role in determining MP levels at harvest.

A higher frequency of days over 30 °C during the growing season is one consequence of climate change [41]. Warming temperatures due to climate change may reduce MP content in later maturing Bordeaux cultivars typically grown in cool climate regions (e.g., Cabernet franc, Cabernet Sauvignon, Merlot, and Sauvignon blanc). As a result, the long-term warming associated with climate change could be of benefit in some regions where the climate is currently marginal for production based on growing degree days, the length of the growing season, or weather during the maturation period. Conversely, longer and/or warmer maturation periods may or may not be beneficial in warmer grape growing regions. In these regions, the MP content may not be sufficient to maintain the varietal character of some wine styles if excessive MP degradation occurs. Thus, climate change mitigation strategies need to adapt to local conditions.

Changes in the climate over the past decades and rising temperatures are closely associated with changes in grapevine phenology [42]. As a result, these temperature changes are creating a shift in phenology, leading to shorter periods between veraison and harvest, and grapes are being harvested earlier compared to historical harvest dates in traditional grape growing regions such as Bordeaux and other European viticultural regions [42,43,44,45]. While this may seem such as an ideal situation, there may be negative effects of reduced MPs with respect to regional identity and the sensory profile of the wine. Specifically, the ‘typicity’ of some regional wines may change as the styles transition away from incorporating some background MP-associated green and vegetal nuances into wines characterised more by fruity, floral, and potentially cooked notes [37,45]. Another potential consequence in some regions may be an unbalanced wine due to a rapid sugar accumulation in the grapes before reductions in MPs occur. Since sugar accumulation and the accumulation and degradation of a secondary metabolite such as MPs are not necessarily correlated [37,44], winemakers may be forced to harvest grapes with suboptimal flavors (e.g., higher MP levels) to achieve acceptable sugar and acid levels when higher alcohol wines are not desired.

Many studies have attributed the vine water status as a component of terroir because it impacts the physiology of the vine and the biosynthesis of secondary metabolites, including aroma compounds and their precursors [44,46,47]. Climate change, particularly the projected temperature increases and changes in precipitation [48], will lead to a change in the water status of the vines during the course of the growing season [49]. Vineyard soils can impact grape aroma compounds, but it is largely due to the physical characteristics of the soil such as the water holding capacity and drainage that influence the vine water status and vine vigor [46,47,50]. Therefore, climate change-associated effects on the water content in soil, water availability, and vine water status impact the vine growth, physiology of the vine, and synthesis/degradation of MPs. Higher water inputs through irrigation or rainfall can increase IBMP and other odorants [20,51]. These effects are usually a result of more vigorous vines and shading of the fruit zone [20,51,52]. Erratic weather, such as extreme precipitation events coupled with warmer temperatures, may increase the vigor and canopy size that could exacerbate the shading of the fruit, leading to higher IBMP levels. However, an increased incidence of drought may have the opposite effect and reduce the canopy size [20,53]; therefore, increasing cluster exposure leading to more degradation of MPs in grape berries [32].

There is also some evidence that MP content, particularly IBMP, can be higher in fruit from warm regions with growing conditions that have higher temperatures and a highwater availability [38]. Harris et al. [38] found higher levels of IBMP in grapes from warmer regions of California (Central Valley) than those in cooler regions of Bordeaux and New York State (Finger Lakes). Therefore, regions with increasing temperatures and higher precipitation may be in a dilemma trying to manage adequate fruit maturity.

### 2.4. Influence of Viticultural Practices

Since MPs are influenced by the cultivar, temperature, and sunlight, research has been conducted to address the management of MPs—as with other secondary metabolites—through viticultural practices [13,51,54]. Several studies have examined the effects of cluster exposure on MP levels. The training system [13], vine spacing [51], row orientation [55], leaf removal [32,54,56], and irrigation [51,57] can all impact MP levels and the potential wine quality through their influence on fruit cluster exposure. Training systems that increase temperature and light exposure both in the canopy and in the fruiting zone have been associated with a lower MP content [13]. The optimal choice of training system will depend on the cultivar, climate, and soil type. High levels of IBMP are commonly found in shaded berries in highly vigorous grapevines [32], but a proper training choice can alleviate excessive fruit shading. For example, divided canopy systems (i.e., Scott-Henry, Lyre or Geneva Double Curtain) can improve the canopy architecture and, as a result, increase the fruit exposure on vigorous vines [58] that may result in lower MPs at harvest. In addition to training systems, vine spacing can impact the shoot density and canopy length [59]. Higher concentrations of IBMP have also been reported in Sauvignon blanc when planted in rows that were orientated east–west compared to north–south [55]. High shoot densities can produce canopies with higher proportions of shaded leaves and fruit [60]. High planting densities can exacerbate vine vigor under these conditions [61] and, as a result, higher planting densities can result in vines with denser canopies, less fruit exposure and a higher MP content in developing berries [51]. Therefore, a proper planting density with increased vine spacing is an important consideration for cool climate regions where vine vigor may be excessive.

Defoliation via leaf removal to expose grape clusters has also become a widely used method in cool climate regions to reduce the MP content in red Bordeaux grape cultivars. Early defoliation can reduce the concentration of MPs in grapes at harvest [32,54], particularly under poor growing conditions such as in cool, wet years [56]. Early leaf defoliation (10–40 days after flowering) reduced the IBMP content by 28–58% at veraison and 34–88% at harvest compared to the non-defoliated vines [54]. The efficacy of basal leaf defoliation depends on the severity and timing of leaf removal, but can also vary with cultivar and vintage [32,54]. Canopy management practices such as defoliation in the fruiting zone are more critical in cooler and/or wet years that can contribute to excessive vine growth and shading of fruit clusters as well as the slower degradation of MPs [32]. Poor viticulture management, including excessive nitrogen fertilisation [52] resulting in shade, or grapes from vigorous vines with a high canopy density, can lead to heighten the IBMP content [18]. Viticultural practices can, therefore, be used to help mitigate effects of climate change in the context of MPs and managing the flavor in the vineyard.

Viticulturists in both warm and cool climate regions may need to modify existing vineyard practices to achieve ideal fruit maturity and to manage MPs—as well as other aroma and flavor compounds—with the changing climate, depending on how the weather is changing and the cultivars they are growing. Climate change will likely increase the need for irrigation in many warm and dry regions because of hotter temperatures and increased evapotranspiration demands in conjunction with reduced rainfall in the winter months or during the growing season [48,62]. Consequently, irrigation will be a critical strategy to ensure adequate water is provided to grapevines for proper growth and development and to achieve a desirable yield and quality attributes [63]. Greater precision in irrigation strategies will also be needed to avoid water applications that may lead to excessive vigor and fruit shading, while concomitantly protecting water resources for sustainability purposes. For example, IBMP levels during fruit maturation can be controlled through proper irrigation strategies [57]. Excessive irrigation or fertigation may increase vine growth and fruit shading that, subsequently, increase MPs; however, deficit irrigation can reduce vegetative growth [64], increase fruit exposure [63], and result in lower MPs during fruit maturation and harvest [57]. Illustrating this, a 67% decrease in IBMP at harvest was achieved through deficit irrigation (70% evapotranspiration demand) compared to full irrigation with nitrogen supplementation in Merlot grapes [57]. Mechanization [65] and precision viticulture technologies are increasing in grape growing regions to improve efficiencies in the vineyard and reduce costs [66,67]. As innovations in vineyard management advance, there is a gap in the knowledge of how these technologies may impact fruit quality and grape/wine flavor, including MP accumulation and degradation, in developing fruit. More research is also needed to determine if the strategies studied to reduce the MP content in wine in colder climates can be adapted to preserve the MP content in warmer climates. Therefore, future studies should take this into consideration as vineyard practices adapt to changing climates. 

One of the mitigation strategies to combat climate change in traditional wine regions may be to utilize new grapevine material or later maturing cultivars [45,68]. For example, regions that currently grow cool climate cultivars that perform ideally under shorter growing seasons with a low number of growing degree days (<1200) may need to be replanted with later maturing cultivars if the growing seasons continue to become warmer and longer. Therefore, a cool climate region that grows Chardonnay and Pinot noir, which are cultivars associated with low MPs, may start transitioning to later maturing ones such as the Bordeaux cultivars Sauvignon blanc, Cabernet franc, or Cabernet Sauvignon with higher MPs. This would then require these regions to potentially adapt their viticultural practices to ensure that the grapes have the desired varietal characteristics but do not have excessive MP levels that can lead to green or unripe flavours.

**Figure 2:**
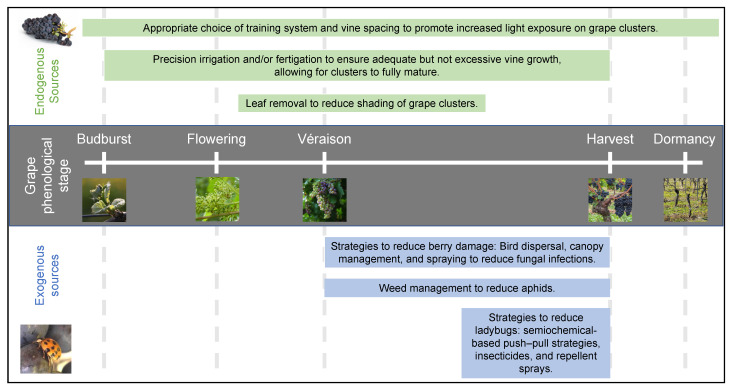
Effective vineyard interventions for managing methoxypyrazine levels by phenological stage.

Figure 2 includes an overview of the key interventions for managing grape-derived MPs in the vineyard through the different phenological stages.

## 3. Exogenous Methoxypyrazines

### 3.1. Coccinellidae

In addition to direct impacts of climate change on MP levels from altered grapevine physiology and growth, a warming climate may also prove beneficial to the distribution and survivability of invasive Coccinellidae (‘ladybeetles’), particularly *Harmonia axyridis*, variously known as the multi-coloured Asian lady beetle (MALB) and the harlequin ladybird. When *H. axyridis* beetles from the vineyard are incorporated in with the grapes during harvesting operations, the MP component of their haemolymph [69] can affect both the juice and subsequent wine. Approximately 1.3–1.9 beetles/kg grapes are sufficient for these exogenous MPs to become perceptible [70,71,72] and confer characteristics to the juice or wine known as ladybug taint [73]. Thus, products affected are typically described by terms such as peanut, green pepper, and vegetal [73,74]. The tainting of wine with MPs derived from *H. axyridis* is believed to occur in many global wine regions, including those in North America and Europe [75], but may often be misattributed to grape-sourced MPs, leading to potentially misguided preventative measures being applied in the vineyard. 

Vineyards located near soybean or grain crops appear to be especially vulnerable to aggregation of *H. axyridis*, at least in North America. There, *H. axyridis* will typically feed on their preferred aphid species in these adjacent fields and migrate into vineyards once these crops are harvested [76,77]. As well as providing shelter, grapevines with damaged fruit also provide a secondary food source for the beetles during fall as they seek to build up their reserves for overwintering [78], although they are not believed to directly damage the grapes themselves [76]. Very high densities of beetles can be observed in vineyards around harvest in some years, with the potential for a major impact on wine quality across entire wine regions, as demonstrated, for instance, in Ontario in 2001. 

The MP composition of wines affected by *H. axyridis* differs from that of endogenously sourced MPs. While IBMP is the predominant MP in grapes [75,79], *H. axyridis*-affected wines have higher concentrations of IPMP than IBMP [80,81,82]. Indeed, a ratio of IPMP to IBMB greater than one has been proposed as a ‘diagnostic’ for determining that the greenness in a wine is due to *H. axyridis* rather than grape-derived MPs [75]. Additionally, while SBMP and possibly other MPs from *H. axyridis* haemolymph may contribute to it, IPMP is the dominant ‘green’ odorant in these wines [83]. *H. axyridis* has been shown capable of increasing the IPMP concentration by up to 45 ng/L in research wines [84]. 

Coccinellidae species other than *H*. *axyridis* also contain MPs, specifically *Coccinella septempunctata* (‘seven-spot’) and *Hippodamia convergens* [85,86]. Although the addition of *C. septempunctata* beetles to harvested grapes may lead to an increase in IPMP [80,83] and produce similar sensory profiles to MALB [83] in wine under experimental settings, there are no reports in the literature of *C. septempunctata* or *H. convergens* reaching sufficient densities at harvest to impact the subsequent wines.

### 3.2. Climate Change and Coccinellidae

**Figure 3:**
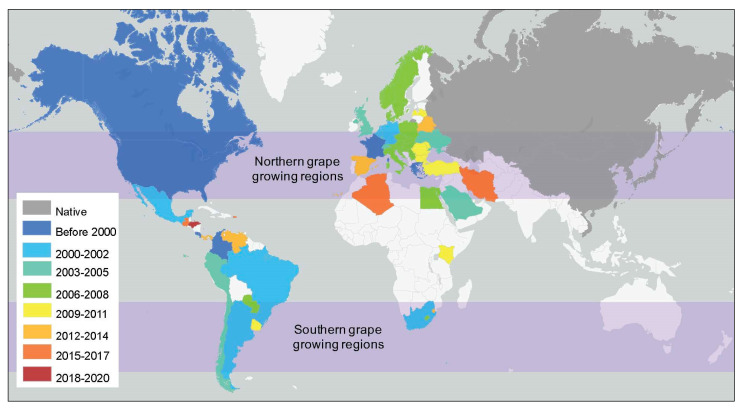
The expanding global distribution of *Harmonia axyridis*. Countries are shaded to indicate when *H. axyridis* was first identified. Readers are referred to Table S1 for primary data sources [21,88,89,90,91,92,93,94].

*H. axyridis* is native to Asia, but intentional introductions as a biocontrol agent for aphids have allowed *H. axyridis* to expand its range worldwide (reviewed in [21]). *H. axyridis* can now be found widely across temperate climates in North America, South America, and Europe [21], with well-established populations in several wine growing regions (Figure 3, Table S1). Understanding the impact of temperature changes associated with climate change on the distribution of *H. axyridis* is important, as temperature impacts the survival of *H. axyridis* over its entire life history [87]. 

Two methods (CLIMEX model and Maxent) have been used to predict the potential range of *H. axyridis* by comparing the climate of a region to the climate-relate responses of the beetle (e.g., optimal temperature and moisture levels for proliferation) [95,96]. The predicted distribution of *H. axyridis* closely matches the actual distribution in colder climates (North America and the United Kingdom [95,97]), but is less successful in warmer climates [95,96]. Although *H. axyridis* does not have established populations in Australia or New Zealand, and is not widely established across Africa [21], the CLIMEX model suggests that the current climate in these winegrowing regions is also suitable for *H. axyridis* [95]. The CLIMEX model has also been used to predict future distributions of *H. axyridis* in Europe under two climate change scenarios in the year 2050. Under a low emissions scenario, ecoclimatic index (EI) values increase in several countries (Belarus, Latvia, Lithuania, parts of Germany, Northern France, Belgium, and the United Kingdom), suggesting that the climate is expected to become more suitable for *H. axyridis* [97]. Under the medium emissions scenario, additional countries are also expected to have increasingly suitable climates for *H. axyridis* (Poland, Ukraine, Russia, and the Netherlands) and invasion/proliferation into new regions is expected to occur more quickly [97]. Thus, climate change in several wine growing regions across Europe is likely to increase the risk of *H. axyridis* vineyard infestation during harvest. 

*H. axyridis* is a multivoltine species capable of producing up to four generations in a single season [98,99,100,101]. Interestingly, in Great Britain and Denmark, which are at the northern limit of the current distribution [21], two generations of *H. axyridis* are typically observed each year, but it has been suggested that a third generation could be possible in warmer years [100,101]. This speculation is supported by the CLIMEX model of Evans and Simpson [97], who predicted that 3-4 generations of *H. axyridis* would be possible in southeastern England by 2050 as a result of climate change. Interestingly, in 2007 and 2008 in eastern England, *H. axyridis* populations increased slowly from April to July, before a rapid increase in the population between July and September and peak population numbers in September–October [102]. As *H. axyridis* can survive long enough for multiple generations to overlap [98,99], these results suggest that the abundance of *H. axyridis* could increase even further if a third generation was observed under climate change. Importantly, this could substantially increase the potential number of *H. axyridis* beetles inside grape clusters at harvest. Thus, climate change will likely create conditions that necessitate more careful vineyard monitoring and management to ensure that *H. axyridis* populations at harvest are below the thresholds for preventing ladybug taint [11]. More research is required to determine if/how the number of generations of *H. axyridis* are impacted by climate change at the southern limit of its European distribution.

It should be noted that the CLIMEX model assumes that the distribution of a species is solely attributable to the climate of a region [95,97]. However, several other factors can impact the distribution of *H. axyridis*, including the availability of resources, competition from native species, the type and quality of the habitat (e.g., urban, agricultural, natural) [21,103], and genetic differences between different populations [104]. Some of these factors are also likely to be impacted by climate change, further complicating the task of predicting both the distribution and abundance of *H. axyridis* in the future. While climate change-related warming will likely expand the potential range of *H. axyridis* towards the poles, other factors may prevent *H. axyridis* from successfully invading these areas. Thus, it is likely that the impacts of climate change on *H. axyridis* in vineyards and their contribution to MPs in wine will vary locally, and more research is needed to understand and mitigate the potential impacts.

### 3.3. Managing Coccinellidae in the Vineyard

The close monitoring of *H. axyridis* densities in vineyards is important, particularly as harvest approaches, so that population densities can be managed to below the level where they can impact the resulting wines. Action thresholds, the density at which *H. axyridis* can be expected to affect juice and wines and, thus, interventions, are indicated [70], vary with the grape cultivar, but the literature converges around an estimate of 1300–1900 beetles/t grapes [70,71,72]. Pickering et al., [72] suggested a more conservative value of 200–400 beetles/t to help account for variability due to cultivar, wine processing factors, and individual sensitivities. Various sampling approaches to assess beetle densities were examined, with binomial sampling reported to be the most accurate [105].

Given that *H. axyridis* feed on damaged grapes [78], good general vineyard management practices that reduce the prevalence of damaged fruit can help. These include sound canopy management, the use of antifungal agents when appropriate, and bird displacement measures [11]. In addition to good general management practices in the vineyard, several interventions targeted at Coccinellidae have been examined in the literature with varying degrees of success reported. Pickering and Botezatu [11] provided a thorough review of the research in this area; here, we examine those interventions that have been shown to be effective in the field. 

#### 3.3.1. Semiochemical Push–Pull Approaches

Semiochemicals are chemicals or mixtures of chemicals that are released by organisms and affect the behavior of other individuals, either from the same species or from another [106]. Semiochemical-based push–pull strategies seek to manage the abundance and distribution of beetles within a vineyard by using a combination of both a repellant and an attractive stimulus. The repellant component ‘pushes’ the beetles away from the grapes, while the attractant ‘pulls’ the beetles to other areas of the vineyard, such as where trapping zones are located.

Several candidate attractants for *H. axyridis* derived from aphids, coccinellids, and nettles have been examined, including Z,E-nepetalactone, [E]-β-farnesene, α-pinene, β-pinene, [-]-β-caryophyllene, IBMP, IPMP, and SBMP, as well as the grape breakdown/fermentation compounds ethanol, acetic acid, acetaldehyde, and isobutanol [107,108]. Of those that have been field-tested, Z,E-nepetalactone appears most effective (tested in a potato field; [108]). Ethyl acetate and a mixture of acetic acid plus acetaldehyde have been reported as repellant to *H. axyridis* [109], but their efficacy under field conditions has yet to be demonstrated. Thus, while semiochemical-based push–pull approaches show some promise, more research under ecologically valid vineyard conditions is needed to elaborate on these findings. 

#### 3.3.2. Spraying

Two general categories of sprays have been investigated—insecticides and non semiochemical-based repellents—and both have demonstrated good efficacy against *H. axyridis.* Within North America, insecticides are a common approach for controlling Coccinellidae within vineyards. Products based on malathion, cypermethrin, dinotefuran, clothianidin, and permethrin are all used, although what is permissible may vary between jurisdictions, as does pre-harvest intervals. The latter is particularly important, as beetles are known to reinfest vineyards on multiple occasions around harvest. Cypermethrin-based sprays such as Ripcord™ 400 EC have been reported as having an extended repellency effect, and both Cypermethrin- and malathion-based products (e.g., Malathion 85 E) show good knockdown success [107]. As *H. axyridis* is capable of contributing MPs and tainting wine up to three days after death [82], winegrowers need to be careful that dead beetles are not also harvested with the fruit at vintage. Indeed, Glemser et al. [110] have reported dead beetles stuck within clusters between grapes after some spray applications.

The application of sulfur dioxide (SO_2_)—a ubiquitous compound used in winemaking—to vines looks very promising as a repellant, with a reduction in beetle density of 50-60% reported at a spray rate of 10 g/L potassium metabisulfite (KMS; [107]). However, it is important—as with a good spraying practice in general—that the SO_2_ is not applied under strong wind conditions, as this may adversely affect its efficacy [110]. Additionally, bentonite (Biobenton)—and garlic powder + KMS—(Buran)-based products have also demonstrated efficacy as vineyard sprays, reducing the density of *H. axyridis* beetles by 39 and 34%, respectively [110]. However, in contrast with SO_2_, the possible effects of these products on juice/wine composition and quality remain to be determined.

#### 3.3.3. Removing Beetles after Harvest

Removing beetles from harvested grapes prior to further processing can also be effective at reducing densities to below those that can taint the wine. Shaker tables are employed in several regions to facilitate this, and anecdotally have been reported as very effective, particularly those models that are designed specifically for Coccinellidae. However, they are typically limited to hand-harvested fruit and the need to process high volumes of grapes in a short period can be a limitation [111]. A newer innovation is that of optical sorters, which can be deployed on grape harvesters. Optical sorters incorporate high-speed cameras and image-processing software to distinguish between and separate grapes from beetles (and other material) and have been reported anecdotally to be effective. There are also reports of wineries immersing harvested grapes in water and allowing for beetles to rise to the surface where they can be removed [111]. However, the impact of this approach on grape sugar concentration (possible dilution effect) and other quality parameters remains to be determined.

## 4. Remediating Methoxypyrazines in the Winery

Whether excessive MPs are derived from grapes or from Coccinellidae, vintners are interested in what can be conducted in the winery to reduce their negative impact on wine aroma and flavour. Several studies have examined how traditional winemaking processes and novel approaches might be used to remediate MPs, and a summary of the main findings is presented in Table 1. Pickering and Botezatu [11] recently provided a thorough review on how winemaking practices impact MP levels, with a focus on IPMP and ladybug taint. Here, we present and discuss the most effective and promising winery interventions for remediating excessive MP levels in wine.

For white wine, settling and clarifying the pressed grape juice is one of the first processing options considered in winery. This is a minimum-intervention step with reasonable effectiveness at reducing the MP load. Both naturally settled and bentonite-clarified juice show significant reductions in IPMP; up to 50%, with higher reductions reported in juice settled for 48 hrs compared to 24 hrs [112]. Most MPs are extracted during the first 24hrs of fermentation; thus, alcohol is not critical to their extraction into the must/wine [13,131]. However, minimizing skin contact where possible is an important consideration, given that the skin contains most of the MPs found in grapes [24]. Similarly, in the case of red wines, shorter maceration times and gentle pressing have been advocated to reduce MP extraction [13]. 

The application of heat to red grape must through both thermovinification or modifications, thereof based on the same principles, has been effective in several studies. Originally employed as a method for increasing the color and phenolic extract during thermovinification, musts are heated for a short time to 60–80 °C. This heating regime has been shown to reduce IBMP in red wines by 29–67% [113], and IBMP, SBMP, IPMP, and DMMP by more modest amounts in Pinot noir wine [80], although the introduction of ‘cooked’ aromas and flavours is a potential limitation [113]. Flash détente (‘flash release’, ‘flash extraction’, ‘Thermoflash’) is a modification of thermovinification, which aims to increase colour and tannins [132]. With flash détente, must from crushed grapes is heated to approximately 85 °C (185 °F), and then transferred to a high-vacuum chamber where the temperature is decreased rapidly. This results in the cell walls of the skin vacuoles to burst, releasing phenolics, anthocyanins and odorants. The water that evaporates during this process is run through a condenser and can be added back to the must as/if required [132]. Flash détente has been reported to reduce the IBMP content in Cabernet Sauvignon wine from 19 to <1 ng/L [114] and to reduce MP-related greenness [133]. Similarly, a marked reduction in the IBMP content of Zinfandel must has been reported after flash détente treatment [134]. An apparent limitation is that these data are not yet reported in peer-reviewed journals, nor has there been a comprehensive report on the impacts of flash détente on the sensory profile of the wines; but, nonetheless, the technology appears to hold considerable promise for reducing MP loads to below threshold levels. 

The use of oak in winemaking is ubiquitous in all wine regions; however, there has been very limited research on how it impacts MPs. Pickering et al. [120] showed no change in IPMP concentration in white or red wine treated with oak chips. However, they noted a significant decrease in several MP-associated green attributes, which was attributable to a sensory masking effect by the oak, in agreement with anecdotal reports from the industry on the effects of barrel aging of commercial wines. While obviously not applicable to all wine styles for stylistic reasons, the judicial use of oak may in many instances ‘do enough’ to hide lower levels of MPs, as can blending with a wine of a lower MP concentration when volumes permit.

Two polymers—silicone and polylactic acid—have been trialed as ‘additives’ to juice and wine on a research scale and demonstrate good efficacy for multiple MPs. For instance, Ryona et al. [126] showed reductions in IBMP and IPMP of 53–93% across a range of white, red, and rosé wines after silicone was added to juice/must. However, a reduction in other odorant compounds was noted, highlighting the need for a sensory evaluation of the wines. Subsequent studies have reproduced the efficacy of silicone when added to wine, with IPMP and IBMP reduced by 96% and 100%, respectively, in Botezatu and Pickering [127] and IPMP, IBMP, and SBMP reduced by 38%–44% in Botezatu et al. [128]. The latter authors also conducted a sensory evaluation of the wines; however, the sensory impact of silicone treatment was not clearly elucidated. 

Polylactic acid also holds promise. It has the advantage of being a biodegradable, compostable polymer produced from renewable sources [135] and can be configured in multiple ways to integrate with wine processing systems, such as a solid tank insert, a filtration component, or pellets added directly into juice/wine and later removed [128]. While reductions in IBMP, SBMP, and IPMP in wine of between 36% and 78% have been shown [127,128] with minimal effect on other wine aroma compounds [128], the extent to which the sensory characteristics of the high-MP wines are improved is unclear [128]. Additionally, the efficacy of both silicone and polylactic acid are yet to be demonstrated at a commercial scale. Finally, synthetic polymer corks are used commercially to close many wines, and have been shown to adsorb IPMP, IBMP, and SBMP [125], leading to MP reductions of up to 21% in bottled wines [119].

Several newer technologies that demonstrate high specificity for MPs are very promising but are yet to reach regulatory and/or commercialization stages. High specificity is important, as it implies that desirable odorants, tastants, and pigments in the treated wine will be minimally affected. Of these, odorant-binding proteins and imprinted polymers show particular promise. The odorant-binding protein mMUP2 has been shown to bind with IBMP and IPMP with a very high specificity, and can remove >99% of these MPs when applied to juice, with the protein–MP complex, subsequently, removed with bentonite fining and filtration through a polyethersulfone membrane [123]. Its performance, however, is yet to be demonstrated in the more challenging wine matrix. Finally, recent work has demonstrated that magnetically imprinted magnetic polymers can reduce IBMP by up to 40% in model and white wine and 74% in red wine [129,130]. Because these imprinted polymers incorporate iron oxide nanoparticles, they can be removed from the matrix after treatment with a magnet, and they are reusable for up to five cycles [129].

## 5. Conclusions

MPs are an important class of odorants impacting the quality of wine produced from a range of *V. vinifera* cultivars grown worldwide. They are sourced from both grapes and an incorporation of Coccinellidae beetles in harvested fruit. Climate change has and will continue to have a profound influence on viticulture, with impacts on grapevine development and physiology affecting secondary aroma compounds, including MPs. Warming conditions may benefit cool climate regions growing grapes with a high MP content, but challenges such as extreme weather and excessive precipitation may negate some of these benefits. Regions that are already considered warm are likely the areas that will be most significantly impacted where the sustainability of grapevine production may be threatened, aside from grape and wine flavour considerations. In these regions, new adaptation strategies such as increased irrigation and the use of new grapevine material will be required to mitigate climate change. Climate-related factors also play a role in the expansion of *H. axyridis* into winegrowing regions, with future impacts on MP loads in wine likely to vary at both the micro and macro scale. A significant body of research exists to inform optimal approaches for managing this invasive species in the vineyard, with several spray options offering the best protection at present. Multiple remediation options for grape juice and wine with elevated MP levels exist, but vary significantly in their relative efficacy and current commercialisation status. Juice clarification, the heat treatment of must and use of oak appear to be the most effective current options, with several technologies in development potentially offering a greater specificity. Further applied research is encouraged to assist grape growers and vintners in managing MPs effectively in the future.

## Figures and Tables

**Table 1:** Summary of potential winery interventions for remediating methoxypyrazines (MPs) or MP-related greenness in grape juice and wine. ND, not determined or reported; LBT, ladybug taint; Mod., moderate.

Type of Intervention	Treatment or Intervention	Matrix	Major Compounds Targeted or Measured	Main Findings/ Limitations	Efficacy/ Potential	Citation(s)
Clarification	Clarification with bentonite or natural settling	Juice	IBMP	-Up to 50% reduction after 24hrs settling -Cannot be applied to wines requiring skin contact	Mod.	[24,112]
Heat and oxygen	Thermovinification	Juice	IBMP	-A 29–67% reduction -Leads to cooked aromas and flavors	Mod.	[80,113]
	ThermoFlash/ Flash Detente	Juice, must—various varietals	IBMP	-Reductions of up to 95% reported -Reduction in vegetal notes in wines -Limited to red wines -Data do not appear to have been peer-reviewed	High	[114]
	Micro- oxygenation	Wine	ND	-Reduction in MP-related notes -Some reports of increase in vegetal attributes -Not clear if effects are due to MP reduction or perceptual masking -Limited to red wines -MPs were not quantified	Fair	[115,116,117,118]
Packaging	Closure and packaging type	Wine spiked with IBMP, SBMP, and IPMP	IBMP, SBMP, IPMP	-Tetra Pak was most effective at reducing all three MPs (up to 41% for IPMP) -Synthetic closures also led to reductions in MPs (up to 21% for IPMP) -Tetra Pak is not a common packaging option for wines	Mod.	[119]
Radiation/irradiation	Light and UV light	Wine affected by LBT	IPMP	-No effect	Poor	[120]
	Irradiation at 100 Gy (cobalt-60 source)	Wine tainted with LBT	ND	-Improvement in MP-related sensory characteristics reported -Potential for free radicals generated to adversely impact wine quality -Data do not appear to have been peer-reviewed	Low	[121,122]
Fining and additives	Selected yeast strains used for fermentation	Juice spiked with IPMP	IPMP	-Lalvin BM45 increased IPMP by 45% -Lalvin D80 produced wines with high MP-related sensory attributes -Lalvin D21 produced wines with lowest MP-related sensory attributes	Poor	[10]
	Activated charcoal, bentonite	Wine affected by LBT	IPMP	**Activated charcoal** -Reduced IPMP by 34% in white wine -MP related attributes did not change in white wine. -In red wine, asparagus and bell pepper flavor reduced **Bentonite** -No effect on IPMP -Reduced asparagus/bell pepper flavor in red wines	Low	[120]
	Oak chips	Wine affected by LBT	IPMP	-Neither oak chips nor deodorised oak chips affected IPMP concentrations. -Oak chips reduced MP-related sensory attributes in both red and white wine (masking effect)	Mod.	[120]
	Odorant-binding proteins (OBP)	Juice	IPMP, IBMP	-mMUP2 applied to juice and, subsequently, fined with bentonite and filtered with a 10 kDa polyethersulfone membrane removed >99% of IPMP and IBMP -No reports of efficacy in wine -Not yet commercialised	High	[123,124]
Polymers	Natural and synthetic closures added to wine	Wine spiked with IBMP, SBMP, and IPMP	IBMP, SBMP, IPMP	-All closures led to MP reductions -Synthetic closures were most efficient (70–89% MP reduction) -SBMP was most affected -Impact on non-target compounds not determined -Limited commercial application	Mod.	[125]
	Silicone added to juice	Model juice, grape juice, and must	IBMP, IPMP	-IPMP reduced by 93% after 48 hrs. -IBMP reduced by 90% after 40 hrs. -IPMP and IBMP also decreased in control wines -Some non-target volatile compounds decreased with treatment	High	[126]
	Plastic polymers added to wine	Wine spiked with IBMP, SBMP, and IPMP	IBMP, SBMP, IPMP	-Polylactic acid reduced IPMP by 52% and IBMP by 36% after 24 hrs. -Silicone reduced IPMP by 96% and IBMP by 100% after 24 hrs.	Mod. (PLA) to High (silicone)	[127]
	Polylactic acid and silicone added to wine	Wine spiked with IBMP, SBMP and IPMP	IBMP, SBMP, IPMP	-Reduction of 38–44% in MPs for silicone polymer -Reduction of 75–78% for MPs for polylactic acid polymer -Minimal impact on other volatile compounds -Sensory impacts were not clear, and generally showed minimal effect from the treatments	Fair	[128]
	Magnetic polymers (molecularly imprinted (MIMP) and non-molecularly imprinted (N-MIMP))	Wine spiked with IBMP	IBMP	-MIMP reduced IBMP by 45% after 30 minutes of contact -N-MIMP reduced IBMP by 38% after 30 minutes of contact -Magnetic polymers are recoverable and reusable -Not yet commercially available	High	[129]
	Molecularly imprinted magnetic polymers and polylactic acid (PLA)	Grape must spiked with IBMP, pre- and post-fermentation	IBMP	-Pre-fermentation MIMP led to 30–40% reduction in IBMP -Post-fermentation MIMP led to 74% reduction in IBMP -Post fermentation PLA led to 18% reduction in IBMP -MIMP led to reduction in “fresh green” aromas in wines -Not yet commercially available	Fair (PLA)–High (MIMP)	[130]

## Data Availability

Data used to generate Figure 3 are included in the Supplementary Materials.

## Supplementary Materials

The following are available online at https://www.mdpi.com/article/10.3390/biom11101521/s1, Table S1: primary sources used to generate the distribution of *Harmonia ayridis* (Figure 3).
